# Serum osmolarity as a predictor of mortality in ICU COVID-19 patients: a retrospective analysis

**DOI:** 10.7717/peerj.20590

**Published:** 2026-01-12

**Authors:** Mehmet Toptas, Özlem Dikme, Ozgur Dikme, Abdurrahman Tünay, Mensure Yilmaz Cakirgoz, İbrahim Akkoç

**Affiliations:** 1 Department of Anesthesiology and Reanimation, İstanbul Training and Research Hospital, İstanbul, Fatih, Turkey; 2 Department of Emergency Medicine, İstanbul Training and Research Hospital, İstanbul, Fatih, Turkey; 3 Department of Anesthesiology and Reanimation, İzmir City University, İzmir, Bayrakli, Turkey; 4 Department of Anesthesiology and Reanimation, Basaksehir Cam and sakura City Hospital, İstanbul, Basaksehir, Turkey

**Keywords:** Serum osmolarity, COVID-19, Critical Illness, Mortality

## Abstract

**Background:**

Serum osmolarity, reflecting fluid and electrolyte balance, may serve as a prognostic marker in critically ill patients, but its role in COVID-19 is not well established. This study evaluated the association between admission serum osmolarity and in-hospital mortality in critically ill COVID-19 patients.

**Methods:**

We conducted a retrospective study including 267 critically ill COVID-19 patients admitted from the ED to the ICU of a tertiary-care hospital between March 2020 and April 2022. Data on demographics, thoracic computed tomography (CT) findings, vasopressor use, ventilation support, laboratory values, and in-hospital mortality were obtained. Serum osmolarity was calculated using the formula. The primary outcome was in-hospital mortality; secondary outcomes included vasopressor use, endotracheal intubation (ETI), and laboratory parameters. Statistical analyses included Mann–Whitney U and chi-square tests, logistic regression, and receiver operating characteristic (ROC) curve analysis.

**Results:**

Of 267 patients, 203 were non-survivors and 64 survivors (mortality 76%); mean age was 53.8 ± 12.3 years, 59.6% male. Survivors had higher median serum osmolarity (288.37 *vs.* 285.75 mOsm/L, *p* = 0.034) and sodium (Na) (135 *vs.* 133 mEq/L, *p* = 0.004). Sodium demonstrated slightly superior discrimination (AUC = 0.620) compared to osmolarity (area under the curve (AUC) = 0.588). In multivariate logistic regression, serum sodium (OR = 0.89, 95% CI 0.82–0.97), inotropic agent use (OR = 3.73, 95% CI [1.65–8.42]), and endotracheal intubation (OR = 5.20, 95% CI [2.11–12.84]) were independent predictors of mortality. The model’s c-statistic was 0.713 (95% CI [0.654–0.771]) with 70.4% sensitivity and 65.8% specificity.

**Conclusions:**

Lower admission serum osmolarity and hyponatremia were independently associated with increased in-hospital mortality in critically ill COVID-19 patients. Although Na slightly outperformed calculated osmolarity, the latter remains a practical, integrative prognostic tool for early risk stratification. Prospective studies should evaluate whether timely correction of hypo-osmolar or hyponatremic states improves outcomes.

## Introduction

Serum osmolarity is a key determinant of total body water distribution and is governed by the concentrations of serum electrolytes, glucose, and urea. Disturbances in osmolarity are strongly linked to fluid imbalances, such as dehydration and hypernatremia, which primarily result from alterations in water balance but may also arise from other mechanisms, ultimately contributing to adverse clinical outcomes, including increased mortality. Serum osmolarity is routinely used in the diagnostic evaluation of electrolyte and metabolic disorders, providing clinicians with essential information for patient management (Najem et al., 2024). In clinical practice, serum osmolality is most often estimated by automated calculation rather than direct measurement, as it is faster and more practical (Faria et al., 2017). Regulation of hydrolytic balance is routinely assessed in clinical laboratories to aid in the differential diagnosis of renal dysfunction and small molecule poisonings. Serum osmolarity is also useful for evaluating the effects of specific treatments and toxins on an individual’s fluid balance (Martín-Calderón et al., 2015). The first formula for calculating serum osmolarity was introduced by Dorwart and Chalmers in 1975, followed by a simplified version by Smithline and Gardner in 1976, providing clinicians with a practical tool to estimate osmolarity and guide the assessment of fluid and electrolyte disorders (Heavens et al., 2014).

The prognostic value of serum osmolarity has been investigated across various patient populations, with hyperosmolarity consistently associated with increased mortality (Dikme & Dikme, 2016). However, this association has not been thoroughly validated in critically ill patients in intensive care units (ICUs), particularly those with severe respiratory infections such as COVID-19. Experimental studies suggest that hyperosmolarity may aid recovery from lung injury by suppressing cytokine production, and clinical data indicate that hypernatremia is not consistently associated with mortality in patients with respiratory disease (Lucijanic et al., 2023). Nevertheless, the overall role of serum osmolarity as a prognostic marker in this population remains unclear, highlighting the need to evaluate its predictive value in critically ill including Coronavirus Disease 2019 (COVID-19) patients.

Thus, it is important to re-evaluate the association between serum osmolarity and mortality in patients with respiratory illness. In recent years, the world has faced several respiratory virus outbreaks, including COVID-19, which was first reported in Wuhan in December 2019 and declared a pandemic by the World Health Organization on March 11, 2020. As of January 5, 2025, COVID-19 has caused 7,083,246 deaths worldwide (World Health Organization, 2025). Given their higher mortality compared to other respiratory viral infections, early prognostic markers are crucial for managing severe cases (Camacho-Domínguez et al., 2024; Grasselli et al., 2020). While sodium, glucose, and blood urea nitrogen (BUN) are recognized prognostic indicators, the predictive value of combined serum osmolarity in COVID-19 remains uncertain. Therefore, this retrospective study aimed to examine the association between admission serum osmolarity and in-hospital mortality in critically ill COVID-19 patients admitted from the emergency department (ED) to the ICU of a tertiary care hospital between March 2020 and April 2022.

## Materials & Methods

### Data collection

This study received ethics approval from the University of Health Sciences Kanuni Sultan Suleyman Research and Training Hospital Scientific Research Ethics Committee (Decision No. 113, dated 22.05.2024). Due to the retrospective design, written informed consent was waived, although all participants were duly informed.

We retrospectively evaluated COVID-19 patients admitted from the ED of a tertiary-care hospital to the same hospital’s ICU. Patient data were extracted from the Hospital Information Management System (HIMS), a database maintained by the hospital that contains comprehensive health information for all patients. Collected data included age, gender, thoracic computed tomography (CT) results, vasopressor use, ventilation support status (high-flow nasal oxygen (HFNO) or endotracheal intubation (ETI)), in-hospital mortality, and serum laboratory values at ED admission, including sodium (Na), potassium (K), glucose, urea, blood urea nitrogen (BUN), neutrophil count, lymphocyte count, neutrophil-to-lymphocyte ratio (NLR), C-reactive protein (CRP), procalcitonin (PCT), ferritin, and D-dimer.

A total of 267 critically ill patients with confirmed COVID-19 who were admitted to the ICU between March 15, 2020, and April 30, 2022, were included. Patients were excluded if they were pregnant, younger than 18 years, had significant burns at admission (>10% total body surface area), acute intoxication or chronic alcohol abuse (occasional/social use was permitted), regular use of mannitol (*e.g.*, for intracranial pressure control or chronic conditions), treatment with antidiuretic hormone, chronic/home steroid or mineralocorticoid therapy, nephrotic syndrome, chronic hyponatremia or hypernatremia, diabetes insipidus, syndrome of inappropriate antidiuretic hormone, liver cirrhosis, or a concurrent diagnosis of diabetic ketoacidosis or nonketotic hyperosmolar hyperglycemic coma at admission.

### COVID-19 diagnosis

COVID-19 was diagnosed based on compatible clinical symptoms and a positive SARS-CoV-2 polymerase chain reaction (PCR) test, performed on nasopharyngeal or oropharyngeal swab samples collected at the time of ED admission.

### Serum osmolarity calculation

Several equations have been proposed for calculating serum osmolarity, but a validation study identified only five as optimal for accurate prediction (Martín-Calderón et al., 2015). In this study, serum osmolarity was calculated using one of these optimal formulas: Serum Osmolarity (mOsm/L) = [2 ×Na (mEq/L)] + [Glucose (mg/dL) / 18] + [BUN (mg/dL) / 2.8]. The normal range was defined as 275–295 mOsm/L. Only values of serum Na, glucose, and BUN measured simultaneously were used for the calculation. Patients without sufficient data to calculate serum osmolarity were excluded from the analysis. Outcomes were compared among three groups based on serum osmolarity at admission: hypo-osmolarity (<275 mOsm/L), normo-osmolarity (275–295 mOsm/L), and hyper-osmolarity (≥295 mOsm/L).

### Outcomes measured

The primary outcome of this study was the association between admission serum osmolarity and in-hospital mortality. Secondary outcomes included the relationships between admission serum osmolarity and the need for vasopressor support, ETI, and selected biochemical and hematological parameters.

### Statistical analysis

Descriptive statistics were used to summarize patient characteristics. Continuous variables were reported as mean ± standard deviation or, for non-normally distributed data, as median with interquartile range (IQR). Categorical variables were expressed as counts and percentages. Normality was assessed using the Shapiro–Wilk test. Group comparisons for continuous variables were performed using the Mann–Whitney U test, and categorical variables were compared using the chi-square test.

A binary logistic regression model was constructed to identify independent predictors of in-hospital mortality, using the enter method with a significance level of 0.05. Goodness-of-fit was evaluated for all logistic regression models. Receiver operating characteristic (ROC) curves were generated to assess diagnostic performance, with the area under the curve (AUC) reported. All analyses were performed using SPSS version 28.

## Results

A total of 267 patients with confirmed COVID-19 were included, with data obtained from the HIMS database. The mean age was 53.8 ± 12.3 years, and 159 patients (59.6%) were male. The study comprised 203 non-survivors and 64 survivors, resulting in a mortality rate of 76%. All deaths were directly attributable to COVID-19 complications, including severe acute respiratory distress syndrome (ARDS) and multi-organ failure. The high mortality reflects that this single tertiary referral center primarily treated the sickest patients during pandemic surges. Patient characteristics stratified by survival status are presented in Table 1.

**Table 1:** Characteristics of survivors and non-survivors.

	**Survivors**		**Non-Survivors**		***p***
	**Med/n**	**IQR/%**	**Med/n**	**IQR/%**	
**Demographical characteristics**					
Age, years	56	48.5–65	56	47.5–62	0.393^*^
Males	35	54.7	124	61.1	0.363^#^
**Laboratory results**					
Serum osmolarity	288.37	282.56–295.69	285.75	278.27–293.91	0.034^*^
Hypo-osmolar	4	6.2	33	16.3	
Normo-osmolar	43	67.2	125	61.6	0.125^#^
Hyper-osmolar	17	26.6	45	22.2	
Na, mmol/L	135	132–138.5	133	130–136	0.004^*^
Hypo-natremia	29	45.3	126	62.1	
Normo-natremia	31	48.4	75	36.9	0.018^#^
Hyper-natremia	4	6.3	2	1	
K, mmol/L	4.21	3.84–4.93	4.08	3.69–4.62	0.137^*^
Glucose, mg/dL	130.85	114.1–194.15	142.1	112.7–239.25	0.967^*^
Urea, mg/dL	49.20	30.25–67.95	52	36.85–79.55	0.064^*^
BUN, mg/dL	22.96	14.12–31.72	24.27	17.2–37.43	0.197^*^
Creatinine, mg/dL	1	0.8–1.45	1.1	0.8–1.7	0.130^*^
e-GFR, mL/min/1.73m2	72.39	43.34–95.44	64.41	37.53–88.97	0.150^*^
Neutrophils, 10^9^/L	7.98	5.7–12.11	7.12	4.08–10.68	0.038^*^
Lymphocytes, 10^9^/L	1.02	0.8–1.71	0.9	0.58–1.30	0.019^*^
NLR, %	7.78	4.17–12.34	7.36	4.06–12.4	0.880^*^
CRP, mg/dL	145.85	71.94–214.8	117.1	62.95–196.5	0.419^*^
PCT, μg/L	0.21	0.12–0.82	0.35	0.14–1.51	0.048^*^
Ferritin, μg/L	538.75	272–786.6	634.8	322.8–1,143	0.167^*^
D-Dimer, μg/L	1235	680–2,465	1,300	780–2,715	0.398^*^
**Need for Support Therapies**					
(+) Inotropic Agent	17	26.6	148	72.9	<0.001^#^
HFNO	11	17.2	44	21.7	0.439^#^
ETI	31	48.4	186	91.6	<0.001^#^
**CT results**					
Viral pneumonia	57	89.1	170	83.7	0.299^#^
Pleural Effusion	20	31.2	42	20.7	0.081^#^
Consolidation	7	10.9	19	9.4	0.710^#^
Pericardial Effusion	3	4.7	13	6.4	0.614^#^

**Notes.**

* Mann–Whitney-U test.

# X^2^ test.

### Primary outcome

**Figure 1:**
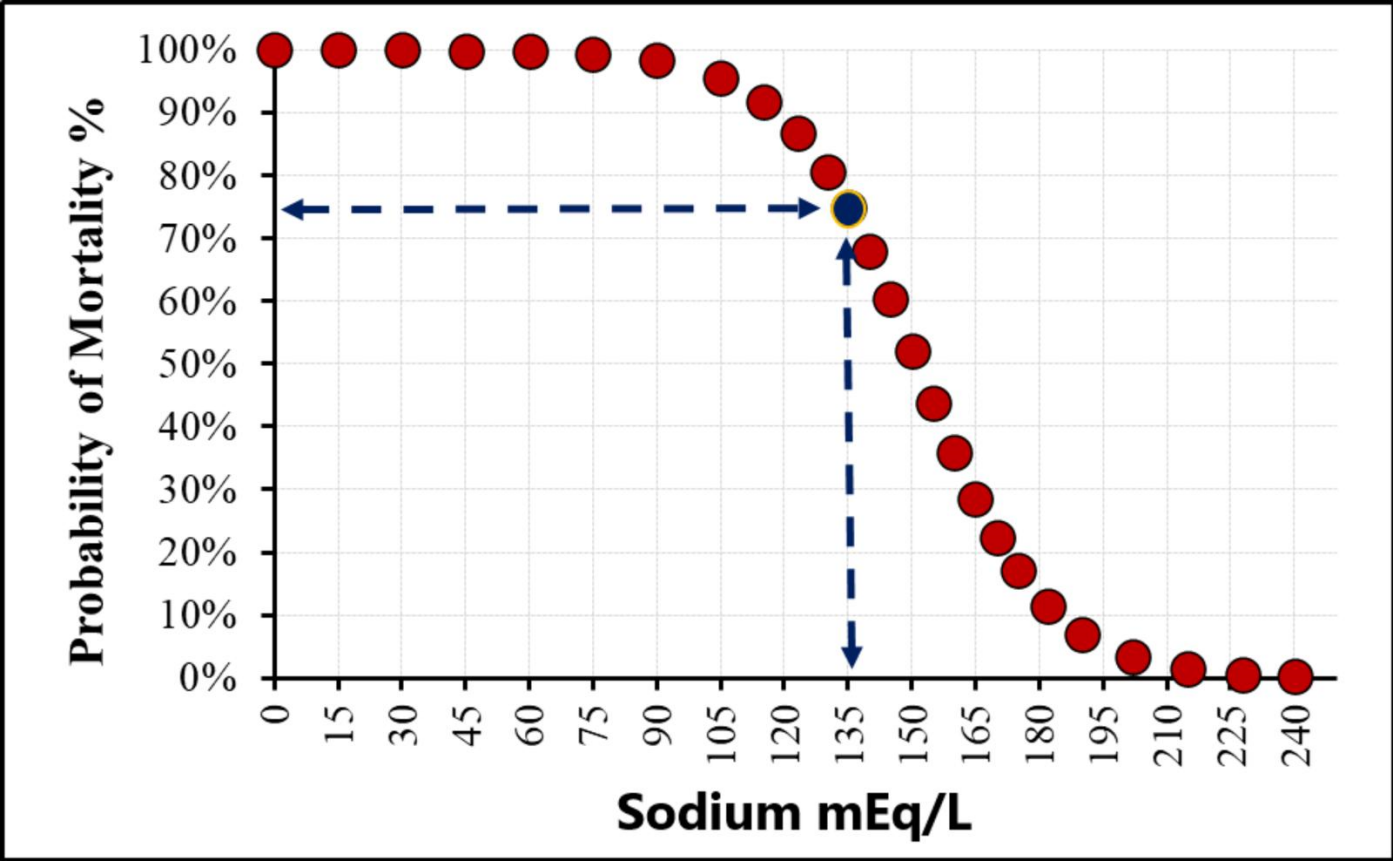
The sodium cut-off value in the study.

Median serum osmolarity and Na levels at admission were significantly higher in survivors compared to non-survivors (288.37 *vs.* 285.75 mOsm/L, *p* = 0.034, and 135 *vs.* 133 mEq/L, *p* = 0.004, respectively). The relationship between sodium (Na) level and the probability of mortality was shown in Fig. 1. These findings suggest that lower serum osmolarity and hyponatremia on admission are associated with increased in-hospital mortality in critically ill COVID-19 patients, highlighting the potential prognostic value of early osmolarity assessment. Notably, ROC analysis demonstrated that serum sodium (AUC = 0.620) slightly outperformed calculated osmolarity (AUC = 0.588) in predicting mortality.

### Secondary outcomes

Median neutrophil and lymphocyte levels were significantly higher in survivors than in non-survivors (7.98  × 10^9^/L *vs.* 7.12  × 10^9^/L, *p* = 0.038; 1.02  × 10^9^/L *vs.* 0.9  × 10^9^/L, *p* = 0.019), whereas the NLR was not associated with mortality. Inflammatory markers including CRP, ferritin, and D-dimer showed no significant association with survival. Although median PCT levels were lower in survivors compared to non-survivors (0.21 µg/L *vs.* 0.35 µg/L, *p* = 0.048), values remained within the range typically observed in viral infections, limiting their clinical relevance.

The need for vasopressor support was significantly lower in survivors than in non-survivors (26.6% *vs.* 72.9%, *p* < 0.001), and survivors were less frequently intubated (48.4% *vs.* 91.6%, *p* < 0.001). Use of HFNO was not associated with mortality. Similarly, lung involvement on thoracic CT scans did not predict mortality: 40 patients (15%) had no involvement, 11 patients (4.1%) had unilateral pneumonia, and 216 patients (80.9%) had bilateral involvement.

### Logistic regression analysis

Results of logistic regression for in-hospital mortality are presented in Table 2. In univariate analysis, admission Na, serum osmolarity, neutrophil count, vasopressor use, and ETI were significantly associated with survival status. Lymphocyte count and PCT levels were not significant. In the multivariate model, Na, vasopressor use, and ETI remained independent predictors of mortality, with corrected odds ratios and confidence intervals as follows: Na (OR = 0.89, 95% CI [0.82–0.97]), ETI (OR = 5.20, 95% CI [2.11–12.84]), and inotropic agent use (OR = 3.73, 95% CI [1.65–8.42]). The c-statistic for the model was 0.713 (95% CI [0.654–0.771]), indicating acceptable discrimination, with a sensitivity of 70.4% and specificity of 65.8%.

**Table 2:** The logistic regression analysis for mortality.

	**Univariate model**				**Multivariate model**			
	**OR**	**CI 95%**		*p* ^*^	**OR**	**CI 95%**		*p* ^*^
Na, mmol/L	0.94	0.89	0.98	0.006	0.89	0.82	0.97	0.008
Serum osmolarity	0.98	0.96	0.99	0.021				
Neutrophils, 10^9^/L	0.95	0.91	1.00	0.048				
(+) Inotropic Agent	0.13	0.07	0.25	<0.001	3.73	1.65	8.42	<0.001
ETI	0.09	0.04	0.17	<0.001	5.20	2.11	12.84	<0.001

**Notes.**

* Logistic regression (Forward LR), Model performance: c-statistic (AUC): 0.713 (95% CI [0.654–0.771]), Sensitivity: 70.4%, Specificity: 65.8%.

### ROC analysis

**Figure 2:**
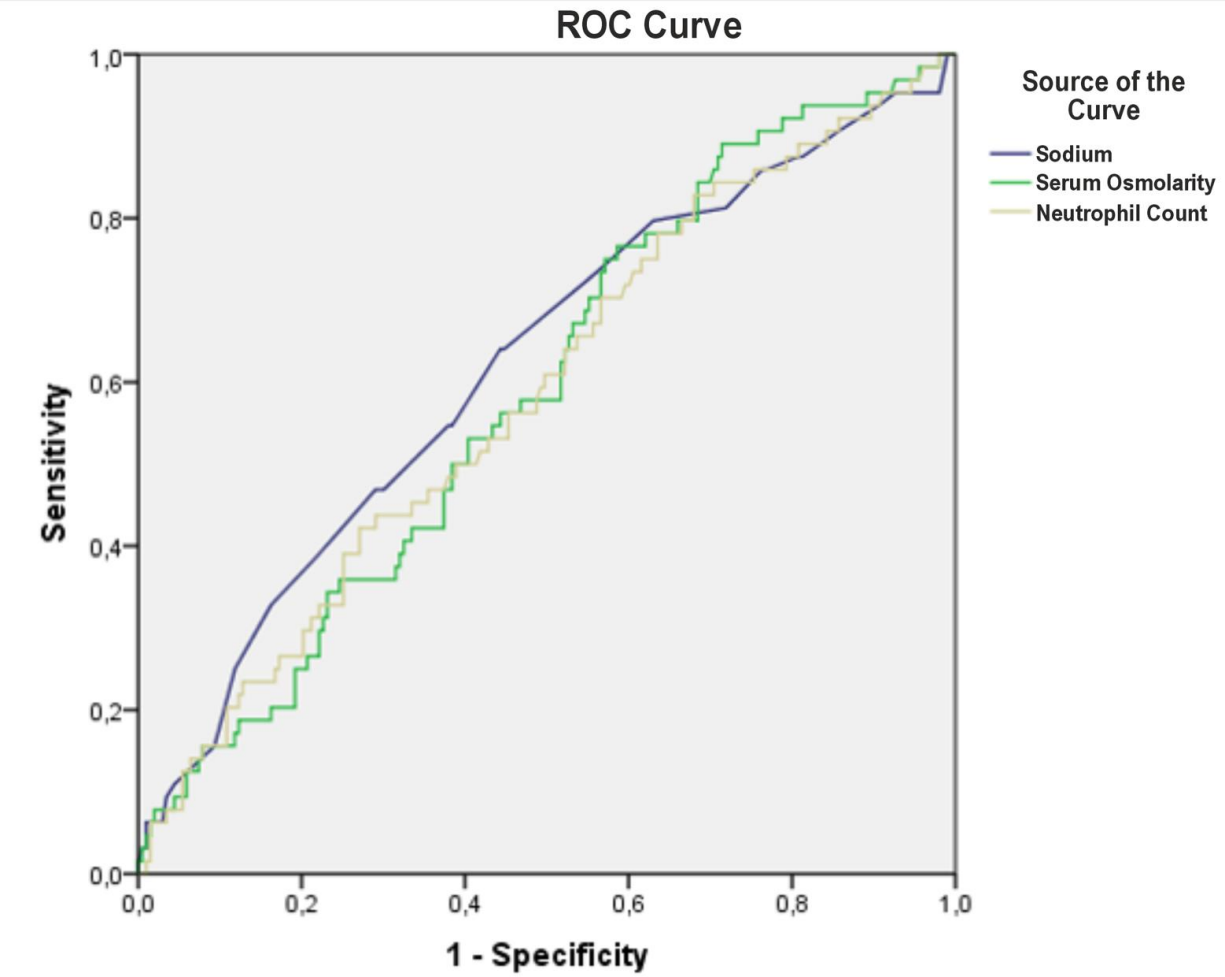
The predictive value of serum osmolarity, sodium and neutrophyl count for mortality.

ROC curve analysis indicated moderate predictive performance for serum osmolarity, Na, and neutrophil count, with AUC values of 0.588, 0.620, and 0.586, respectively. The optimal cut-off value for Na was 135 mEq/L, yielding an AUC of 0.593 (95% CI [0.511–0.675]). At this threshold, sensitivity for predicting mortality was 62.1%, specificity 54.7%, positive predictive value 81.3%, and negative predictive value 31.3% (Fig. 2, Table 3).

**Table 3:** The ROC analysis for mortality.

		**AUC**		**CI 95%**		***p***
**Serum Osmolarity**		0.588		0.511	0.665	*0.034*
**Na**		0.620		0.540	0.701	*0.004*
**Na, cut-off: 135 mmol/L**		0.593		0.511	0.675	*0.025*
**Neutrophyl Count (10** ^**9**^ **/L)**		0.586		0.506	0.666	*0.038*
		**Survivors (n)**	**Non-Survivors (n)**			%
Na (mmol/L)	<135	29	126	Sensitivity		62.1
	≥135	35	77	PPV		81.3
ROC analysis				Specificity		54.7
				NPV		31.3

**Notes.**

ROC Receiver operating characteristic PPV Positive predictive value NPV Negative predictive value AUC Area under the curve CI Confidence interval Na Natrium

## Discussion

In this retrospective study of critically ill COVID-19 patients admitted to the ICU, we found that lower serum osmolarity and hyponatremia on admission were significantly associated with increased in-hospital mortality. Additionally, higher neutrophil and lymphocyte counts were observed in survivors, whereas inflammatory markers such as CRP, ferritin, and D-dimer did not predict mortality. Importantly, vasopressor use, and ETI emerged as independent predictors of mortality, consistent with the severity of critical illness in this study. In the multivariate model, Na, vasopressor use, and ETI remained significant independent predictors, with odds ratios of 0.89, 3.73, and 5.20, respectively, and the overall model demonstrated good discrimination (c-statistic 0.713).

Serum osmolarity plays a central role in intracellular and extracellular water distribution, and disturbances in osmolarity can lead to clinically adverse outcomes. Delayed recognition of osmolar abnormalities or inadequate fluid management may exacerbate these outcomes in critically ill patients. In our study of critically ill COVID-19 patients admitted from the ED to the ICU, lower admission serum osmolarity and hyponatremia were significantly associated with increased in-hospital mortality. While we did not directly assess patients’ volume status, hypo-osmolarity appeared to reflect more severe systemic derangements. Our ROC analysis revealed that serum Na demonstrated slightly better discriminative ability than calculated serum osmolarity (AUC 0.620 *vs.* 0.588), indicating that Na may serve as a stronger individual prognostic factor. Nonetheless, osmolarity, which integrates Na, glucose, and BUN, remains a valuable composite marker reflecting total osmotic balance.

Previous studies have demonstrated the predictive value of serum osmolarity in various patient populations, including patients with sepsis, cardiovascular disease, and non-COVID-19 respiratory illnesses (Dikme & Dikme, 2016; Martín-Calderón et al., 2015). Shen et al. (2017) reported that hyperosmolarity is associated with increased mortality in critically ill patients with cardiac, cerebral, vascular, and gastrointestinal diagnoses. Nicholson, Bennett & Silke (2012) showed that both hypo- and hyperosmolarity on admission were associated with increased mortality in acutely ill medical patients. In COVID-19, Lucijanic et al. (2023) found that both hypo- and hyperosmolar patients had higher mortality compared to normo-osmolar patients, with hyperosmolar patients showing the greatest risk. Our results extend these findings by demonstrating that admission hypo-osmolarity is a significant predictor of mortality among critically ill COVID-19 patients, highlighting its potential as an early prognostic marker. Hypo-osmolarity may identify a high-risk subgroup of ICU patients who could benefit from closer monitoring and early interventions to optimize fluid and electrolyte balance.

Sodium disturbances are a major determinant of osmolarity, and their relationship with mortality is complex. Hypernatremia has consistently been linked to higher mortality, whereas hyponatremia has shown mixed results (Hirsch et al., 2021; Tzoulis et al., 2021; Hu et al., 2021). Hyponatremia is more frequent than hypernatremia, but its prognostic value is limited except in hypovolemic states (Tzoulis et al., 2021). In COVID-19, Hirsch et al. (2021) reported that the association between hyponatremia and mortality was no longer significant after correcting sodium for serum glucose. In our study, hyponatremia was the principal driver of hypo-osmolarity and was independently associated with increased mortality, supporting its value as a prognostic marker.

The logistic regression model yielded a c-statistic of 0.713, with 70.4% sensitivity and 65.8% specificity, indicating acceptable discriminatory performance. These findings reinforce that admission Na levels, vasopressor use, and ETI can serve as reliable indicators of disease severity and mortality risk in critically ill COVID-19 patients. In addition to biochemical parameters, clinical severity markers remained strong predictors of mortality. Vasopressors and ETI during hospitalization were independently associated with death, reflecting hemodynamic instability and respiratory failure in critically ill patients. While inflammatory markers such as CRP, ferritin, and D-dimer were not associated with mortality, median PCT levels were slightly higher in non-survivors but remained within the viral infection range, limiting their clinical relevance. These findings emphasize that combining biochemical markers like osmolarity and Na with clinical severity indicators may improve early risk stratification in the ICU.

Several limitations should be acknowledged. First, the retrospective single-center design may limit generalizability, particularly as our center served as a tertiary referral hospital treating the sickest patients during pandemic surges, which explains the high observed mortality rate. Second, only admission laboratory values were evaluated, and dynamic changes in osmolarity or electrolytes during ICU stay were not analyzed. Third, the study design precludes causal inference, and residual confounding cannot be excluded despite multivariate adjustment. Finally, our sample size, while sufficient to detect meaningful associations, was relatively modest, and external validation in larger multicenter cohorts is warranted.

## Conclusions

This study demonstrates that lower admission serum osmolarity and hyponatremia are independently associated with increased in-hospital mortality among critically ill COVID-19 patients. In the multivariate model, serum Na, vasopressor use, and ETI emerged as significant independent predictors of mortality, with acceptable model discrimination (c-statistic 0.713, sensitivity 70.4%, specificity 65.8%). Among biochemical parameters, Na showed slightly superior prognostic performance compared to calculated osmolarity; however, osmolarity remains an integrated and clinically meaningful indicator of total osmotic and fluid balance.

These findings suggest that early identification and correction of hypo-osmolar or hyponatremic states could contribute to improved management and risk stratification of critically ill COVID-19 patients. Calculated serum osmolarity represents a simple, inexpensive, and readily available biomarker that can be incorporated into initial ICU assessments to guide clinical decision-making. Future prospective, multicenter studies are warranted to validate these results and explore whether targeted interventions to normalize osmolarity and Na levels may improve survival outcomes in this high-risk population.

##  Supplemental Information

10.7717/peerj.20590/supp-1 Supplemental Information 1 Raw data

## Additional Information and Declarations

### Data Availability

The following information was supplied regarding data availability: The raw data is available in the Supplemental Files.
